# Bone Marrow-Derived Multipotent Stromal Cells Attenuate Inflammation in Obliterative Airway Disease in Mouse Tracheal Allografts

**DOI:** 10.1155/2014/468927

**Published:** 2014-09-10

**Authors:** Alicia Casey, Fabian Dirks, Olin D. Liang, Hakima Harrach, Katharina Schuette-Nuetgen, Kristen Leeman, Carla F. Kim, Craig Gerard, Meera Subramaniam

**Affiliations:** ^1^Division of Respiratory Diseases, Department of Medicine, Boston Children's Hospital, Harvard Medical School, 300 Longwood Avenue, Enders 4, Boston, MA 02115, USA; ^2^University of Witten/Herdecke, 58448 Witten, Germany; ^3^Department of Pediatrics, University Children's Hospital, 53113 Bonn, Germany; ^4^University of Münster, 48149 Münster, Germany; ^5^Division of Newborn Medicine, Department of Medicine, Boston Children's Hospital, Harvard Medical School, Boston, MA 02115, USA; ^6^Stem Cell Program, Boston Children's Hospital, Department of Genetics, Harvard Medical School, Boston, MA 02115, USA

## Abstract

Obliterative bronchiolitis (OB) remains the most significant cause of death in long-term survival of lung transplantation. Using an established murine heterotopic tracheal allograft model, the effects of different routes of administration of bone marrow-derived multipotent stromal cells (MSCs) on the development of OB were evaluated. Tracheas from BALB/c mice were implanted into the subcutaneous tissue of major histocompatibility complex- (MHC-) disparate C57BL/6 mice. At the time of transplant, bone marrow-derived MSCs were administered either systemically or locally or via a combination of the two routes. The allografts were explanted at various time points after transplantation and were evaluated for epithelial integrity, inflammatory cell infiltration, fibrosis, and luminal obliteration. We found that the most effective route of bone marrow-derived MSC administration is the combination of systemic and local delivery. Treatment of recipient mice with MSCs suppressed neutrophil, macrophage, and T-cell infiltration and reduced fibrosis. These beneficial effects were observed despite lack of significant MSC epithelial engraftment or new epithelial cell generation. Our study suggests that optimal combination of systemic and local delivery of MSCs may ameliorate the development of obliterative airway disease through modulation of immune response.

## 1. Introduction

Lung transplantation is one of the few treatments available for end-stage lung diseases such as chronic obstructive pulmonary disease, idiopathic pulmonary fibrosis, cystic fibrosis, alpha1-antitrypsin disease, and primary pulmonary hypertension. Five-year survival rates for lung transplantation are significantly lower than other solid organ transplants [1–4], and complications of chronic lung rejection are responsible for the majority of deaths. Chronic lung rejection clinically is termed bronchiolitis obliterans syndrome and pathologically classified as obliterative bronchiolitis (OB). During disease development, chronic inflammatory and fibroproliferative processes lead to small airway obstruction, for which no effective treatment is currently available [5, 6]. OB was initially thought to be caused by immune responses to donor antigens [7]; however, nonimmune mechanisms have also been shown to play an important role in the natural history of the disease [8–10]. Recent studies have emphasized on the role of innate inflammatory cells such as polymorphonuclear neutrophils (PMNs) and macrophages in chronic rejection [5, 11]. PMNs are among the first inflammatory cells to be detected in the bronchoalveolar lavage and lung biopsy specimens of patients with OB [12–14], and they are also increased in the murine trachea transplant model [15, 16]. Macrophages also play an important role in the pathogenesis of chronic rejection as depletion of macrophages ameliorates OB [17]. In addition, a growing body of evidence supports a critical role of lymphocytes in the pathogenesis of OB [5, 11].

Bone marrow-derived multipotent stromal cells (MSCs) have been evaluated experimentally and clinically in the treatment of a wide variety of pathological conditions. Though originally harvested from bone marrow, MSCs have since been isolated from multitude of sources, including adipose tissue, placental tissue, dental pulp, and several others. The paucity of MHC class I and the lack of MHC class II and other costimulatory molecules allow administration of these cells without significant host response [18]. Substantial progress continues to be made with MSCs in lung injury and repair [19, 20]. The ability to repair lung injury was initially hypothesized to be due to the potential ability of MSCs to acquire epithelial phenotype and engraft as structural lung cells. However, engraftment with MSCs, as with most other cell types investigated so far, is a rare event of uncertain physiological significance in lung. As such, emphasis has increasingly shifted toward the profound immunomodulatory, anti-inflammatory, and nonimmunogenic properties of MSCs. In in vitro model systems, MSCs inhibit the proliferation and function of a broad range of immune cells including T cells, B cells, NK cells, and dendritic cells. Notably, MSCs inhibit T-cell proliferation, activation, and cytokine release in response to alloantigens [21]. In addition, MSCs may also affect actions of macrophages [22, 23]. In this context, a number of studies reported the efficacy of MSC administration in various lung injury models in mice, for example, pulmonary hypertension [24], bronchopulmonary dysplasia [25], and OB [26]. Current study is aimed to evaluate different routes of MSC delivery and their respective efficacy using an established heterotopic airway transplant mouse model of OB [27]. Our data indicate that the most effective route of bone marrow-derived MSC administration is the combination of systemic and local delivery. Treatment of recipient mice with MSCs suppressed inflammatory cell infiltration and reduced fibrosis without significant epithelial engraftment.

## 2. Materials and Methods

### 2.1. Animal Maintenance and Heterotopic Airway Transplant Model

C57BL/6 and BALB/c mice (6–12 weeks old) were purchased from Charles River Laboratories (Wilmington, MA). The *β*-Actin/enhanced green fluorescent protein (eGFP) transgenic mice were originally established in Irving Weissman's lab [28]. The mice were housed in the Boston Children's Hospital animal facility under specific pathogen-free conditions. An established model of OB involving heterotopic tracheal transplant with MHC-mismatched combinations of C57BL/6 (H-2b) and BALB/c (H-2d) mice was used as described previously [27]. Briefly, donor BALB/c mice were euthanized and the trachea was resected and immediately placed in ice-cold phosphate-buffered saline (PBS). Recipient C57BL/6 mice were anesthetized and a tracheal graft was inserted into the subcutaneous pocket on the back of the mouse. At 2 days, 1 week, 2 weeks, and 4 weeks after transplantation, the recipient mice were euthanized and the tracheas were harvested immediately. All animal protocols were approved by the Children's Hospital Animal Care and Use Committee.

### 2.2. Bone Marrow-Derived MSC Isolation, Culture, and Differentiation

**Figure 1:**
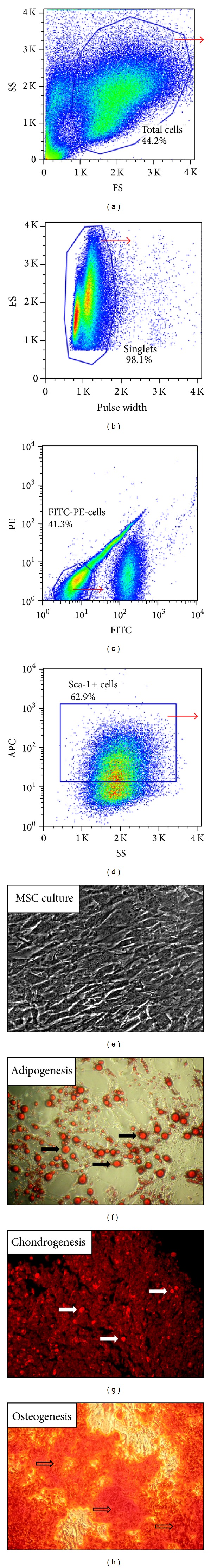
Isolation and trilineage differentiation of mouse bone marrow MSCs. (a)–(d) Representative flow cytometry cell sorting plots show sequential selection of cell populations as indicated by a red arrow: total adherent cells subjected to sorting (a), singlets (b), CD11b-, CD14-, CD19-, CD31-, CD34-, and CD45-cells (c), and Sca-1+ cells (d). (e)–(h) Trilineage differentiation of mouse bone marrow MSCs: Nondifferentiated MSCs in culture (e); black arrows indicate Oil Red O staining of lipid droplets within differentiated adipocytes (f); white arrows indicate staining of Collagen type II (red) produced by differentiated chondrocytes (g); and empty arrows indicate Alizarin Red staining of calcium deposit of differentiated osteocytes (h).

Bone marrow-derived MSCs were isolated from the femurs and tibiae of 6- to 9-week-old C57BL/6 mice and GFP mice as previously described [29]. Briefly, the ends of each tibia and femur were clipped to expose the marrow and then the bones were inserted into adapted centrifuge tubes. The tubes were centrifuged for 1 minute at 400 g to collect the marrow. The pellet was resuspended in 3 mL *α*-minimum essential medium (MEM) medium (Invitrogen) through a 21-gauge needle followed by filtration through a 70-*μ*m nylon mesh filter. The marrow cells were layered on a Ficoll-Paque (Amersham, Piscataway, NJ) density gradient, centrifuged, and plated. The cells were allowed to adhere to the plastic surface of a 25 cm^2^ tissue culture flask (Falcon 3081) for 48 h without disturbance in *α*-MEM medium supplemented with 10% non-heat-inactivated FBS (Hyclone) [30], 10% horse serum (Sigma), 1x L-Glutamine (Invitrogen), and 1% P/S, as described by Peister et al. [29]. Plastic adherent cells were maintained in culture with media changed every 2-3 days. Following 2-3 passages, immunodepletion was performed as per published protocols and the International Society for Cellular Therapy guidelines [31]. The cells were negatively selected for FITC-CD11b, FITC-CD14, FITC-CD19, PE-CD31, PE-CD34, and FITC-CD45, and positively selected for APC-Sca-1 in a fluorescence-activated cell sorter (MoFlo, Beckman-Coulter, Figures 1(a)–1(d)). Fluorescence-conjugated antibodies were purchased from BD Biosciences, BioLegend, and eBioscience. The differentiation potential of MSC cultures was assessed following published protocols [29] (Figures 1(e)–1(h)); Adipogenesis was induced by culturing MSCs in complete *α*-MEM medium, supplemented with 5 *μ*g/mL Insulin, 50 *μ*M Indomethacin, 1 *μ*M Dexamethasone, and 0.5 *μ*M 3-isobutyl-1 methylxanthine (IBMX). After three weeks, the cells were fixed with 10% Formalin and stained with 0.5% Oil Red O in Methanol (Figure 1(f)). For chondrogenesis, StemXVivo mouse chondrogenic supplement and base media (both from R&D Systems) were used. Briefly, 2.5 × 10^5^ MSCs were resuspended in 0.5 mL chondrogenic differentiation medium (base medium plus chondrogenic supplement and penicillin/streptomycin) and centrifuged to form a pellet in the medium. The MSC pellets remained in the culture with fresh medium every 3 days. After three weeks, medium was carefully aspirated and the MSC-pellets were fixed with 10% Formalin, embedded in paraffin, and sectioned. Sheep antibody against type II Collagen and anti-sheep IgG secondary antibody conjugated with NorthernLights 557 (both from R&D Systems) were used to evaluate chondrogenesis of the mouse MSCs (Figure 1(g)). For osteogenesis, cells were supplemented with 20 mM *β*-glycerol phosphate, 50 ng/mL thyroxine, 1 nM Dexamethasone, and 0.5 *μ*M ascorbate 2-phosphate with media change three times per week. At the end of 3 weeks, the cells were fixed with 10% Formalin and stained with Alizarin Red (Figure 1(h)). GFP-MSCs were isolated from GFP mice, which were obtained from Carla Kim's lab (Stem Cell Program, Children's Hospital Boston).

### 2.3. Administration of Bone Marrow-Derived MSCs

Bone marrow-derived MSCs were delivered via different routes prior to allograft transplant. For intratracheal injection (T), 1 × 10^5^ MSCs in 20 *μ*L PBS were injected into the lumen of freshly isolated donor trachea, both ends of which were then carefully tied up with a surgical suture. For intravenous delivery, 1 × 10^5^ MSCs in 100 *μ*L PBS were injected via retroorbital vein (IV). For the combination of these two routes (IV + T) prior to tracheal transplant, 1 × 10^5^ MSCs were given both intratracheally and intravenously. Efficacy of higher doses of MSCs, that is, 2 × 10^5^ (2x) and 10 × 10^5^ (10x), was also examined in our allograft model. PBS was used as control instead of MSCs in all experiments.

### 2.4. Preparation of Tracheal Explants

Recipient mice were euthanized through asphyxiation and implanted tracheas were removed immediately, weighed, and placed on ice. Airways were fixed in formalin-free zinc fixative (BD Biosciences Pharmingen, San Diego, CA), cut in half, and paraffin-embedded with the cut side down. Cross-sections of tracheal tissues (5 *μ*m) were subjected to hematoxylin and eosin (H&E) staining, Masson's trichrome staining, or various specific antibody staining as described below.

### 2.5. Assessment of Tracheal Luminal Obliteration and Epithelial Integrity

H&E staining was performed on all tracheas explanted at 2 days, 1 week, 2 weeks, and 4 weeks after transplantation. Sections were then microscopically examined by two independent investigators who were blinded for the experimental groups. A scale, from 0 (no obliteration) to 4 (maximal obliteration), was used to grade luminal obliteration. Epithelium of the tracheal sections was graded by the percent coverage of the luminal surface.

### 2.6. Immunohistochemistry

Polymorphonuclear neutrophils (PMNs) were stained with rat anti-mouse antibodies to granulocyte marker Ly-6C/6G (Gr-1). Macrophages were stained with rat anti-mouse Mac-3 (BD Biosciences Pharmingen), CD3-positive cells with rabbit anti-mouse CD3 (Abcam), and GFP-positive cells with chicken anti-GFP (green fluorescent protein) antibodies (Aves Labs), respectively. A standard ABC vector kit (Vector Laboratories, Burlingame, CA) and a DAB reagent were used. To visualize CD3-positive cells a TSA Biotin System (Perkin Elmer) was used to enhance staining. Specificity of the staining was confirmed by using isotype control antibody. PMNs were counted manually in the entire section (outside and inside of the tracheal lumen) of the allograft, CD3-positive cells, and macrophages were counted in 4 random high-power fields under a microscope.

### 2.7. Assessment of Fibrosis and Analysis of Hydroxyproline Content

We stained collagen on paraffin-embedded slides to evaluate the extent of fibrosis by light microscopy using a Masson's trichrome staining kit (Sigma) according to the manufacturer's instruction. Tracheal hydroxyproline content was measured to quantify collagen deposition using the method outlined by Woessner Jr. [32]. Briefly, explanted tracheas were manually homogenized in Hanks' balanced salt solution (HBSS) and dried in a speed-vac. Samples were then hydrolyzed in 6 M HCl for 18 hours at 110°C. Aliquots (100 *μ*L) were analyzed for hydroxyproline content by mixing with chloramine-T and Ehrlich's reagent to produce a hydroxyproline chromophore that was quantified at 550 nm spectrophotometrically. Standard curve was generated for each experiment in a 96-well plate using trans-4-Hydroxy-L-proline (Sigma).

### 2.8. Statistical Analysis

The unpaired Student's *t*-test was used for statistical analysis. A *P* value of less than 0.05 was considered to indicate a significant difference between two groups.

## 3. Results

### 3.1. MSCs Suppress Neutrophil Infiltration in the Allografts

**Figure 2:**
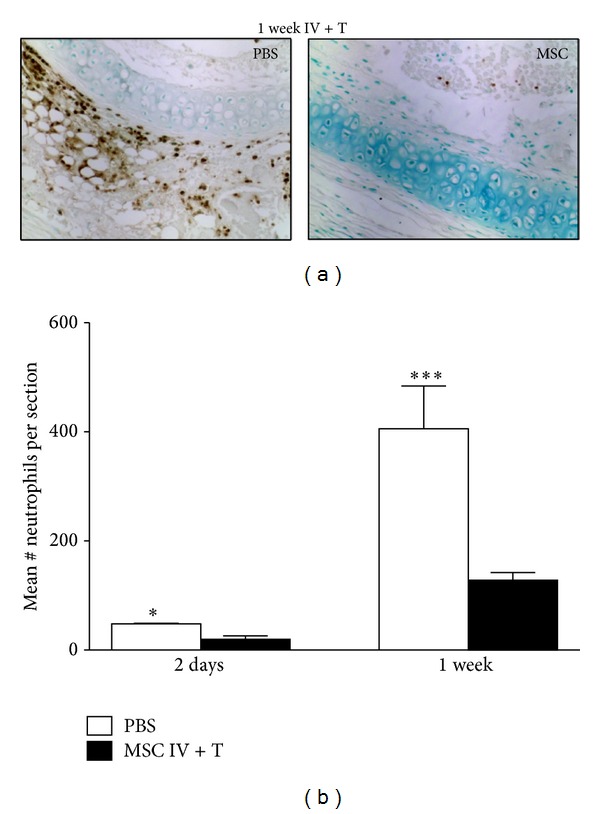
MSCs suppress neutrophil infiltration in the allografts. To assess neutrophil infiltration, donor tracheas from each treatment group were explanted 2 days (PBS, *n* = 3; MSC, *n* = 3) or 1 week (PBS, *n* = 11; MSC, *n* = 8) after transplantation. (a) Representative tracheal sections stained with anti-Ly6C/6G antibodies (dark brown spots, 200x original magnification). (b) The total number of neutrophils per section was counted, and the mean values of 2 days and 1 week after transplantation in the MSC/IV + T-treated group were compared to the respective PBS control group. Significantly less neutrophils were found in the MSC/IV + T-treated group (*P < 0.05; ***P < 0.001). All error bars in (b) indicate SEM.

Histopathological changes in the initial phase of disease development in OB show encroachment of neutrophils as a response to inflammation [5]. To investigate the effects of MSCs in our allograft model, cells stained with granulocyte-antibody Ly6C/6G in the trachea tissue at 2 days, 1 week, 2 weeks, and 4 weeks after transplantation were counted. We observed massive infiltration of neutrophils in the tracheal parenchyma in PBS-control group, whereas the number of neutrophils in the MSC/IV + T-treated mice was significantly decreased in the 2-day and 1-week groups (Figure 2). This inhibition was not significant in the later time points (2-week groups: Control = 255, Experimental = 526, and *P* = 0.6; 4-week group: Control = 34, Experimental = 36, and *P* = 0.9). Interestingly, no significant difference in the number of neutrophils was found in the MSC/IV- or MSC/T-treated mice at any time points (data not shown). These data suggest that MSCs delivered via IV + T could suppress neutrophil infiltration in the allografts during early stage of inflammatory response.

### 3.2. MSCs Reduce Accumulation of Macrophages in the Allografts

**Figure 3:**
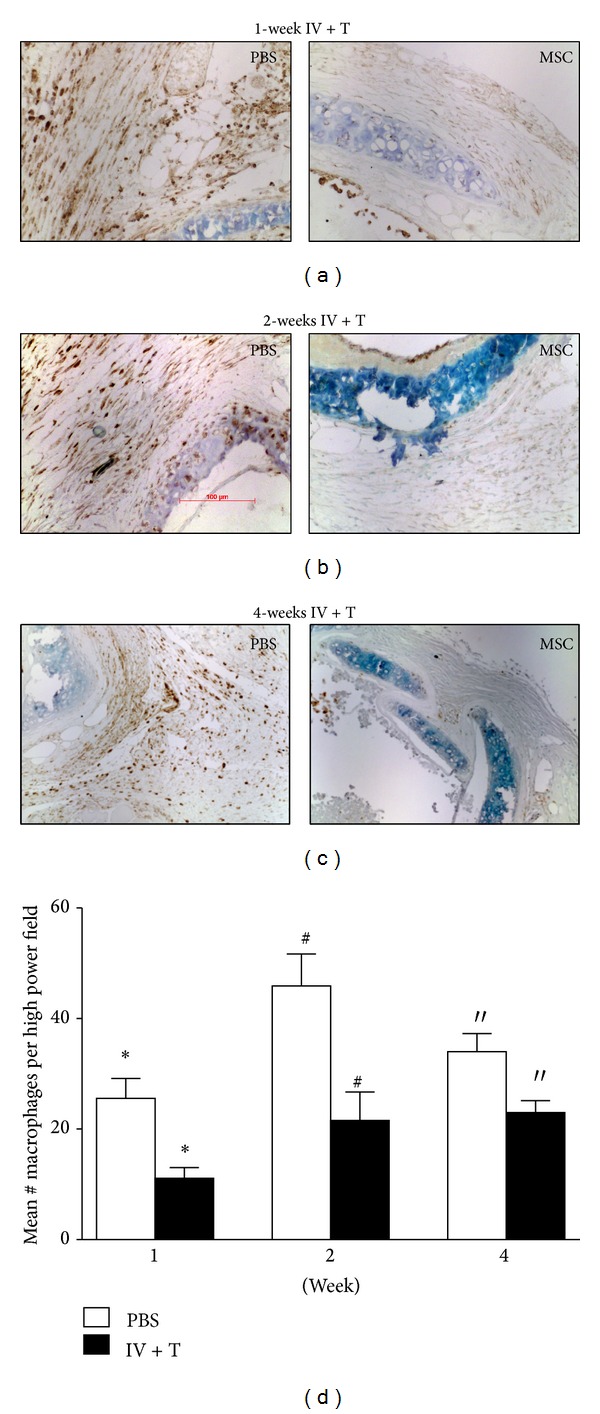
MSCs reduce accumulation of macrophages in the allografts. To assess macrophage accumulation, grafts from each treatment group were explanted at 1 week (PBS, *n* = 5; MSC, *n* = 4), 2 weeks (PBS, *n* = 5; MSC, *n* = 4), and 4 weeks (PBS, *n* = 5; MSC, *n* = 6) after transplantation. (a)–(c) Tracheal sections stained with anti-Mac3 antibodies (dark brown spots) showed reduced numbers of macrophages in the MSC/IV + T-treated group at 1 week (a), 2 weeks (b), and 4 weeks (c) after transplantation when compared to the PBS-treated control group (all representative images 200x original magnification). (d) Macrophages were counted in 4 random high power fields per section. Mean counts of macrophages at 1 week, 2 weeks, and 4 weeks after transplantation in the MSC/IV + T-treated group were compared with PBS-control group. Significantly fewer macrophages were seen in the MSC/IV + T-treated group at 1 week (*P < 0.02), 2 weeks (^#^ *P* < 0.02), and 4 weeks (′′*P* < 0.02). All error bars in (d) indicate SEM.

Similar to our observations on neutrophils, accumulation of Mac 3-positively stained macrophages in the tracheal allograft was significantly reduced in the MSC/IV + T-treated mice at 1 week, 2 weeks, and 4 weeks after transplantation (Figure 3). This beneficial reduction in macrophage accumulation was delayed as macrophage numbers were comparable in the MSC/IV + T-treated group and PBS-control group at 2 days after transplantation. Consistent to what we found with the neutrophils, there was no significant difference noted in the MSC/IV- or MSC/T-treated groups at any time points (data not shown). These data suggest that MSCs delivered via IV + T could reduce long-term accumulation of macrophage in the allografts.

### 3.3. MSCs Inhibit T-Cell Response in the Allografts

**Figure 4:**
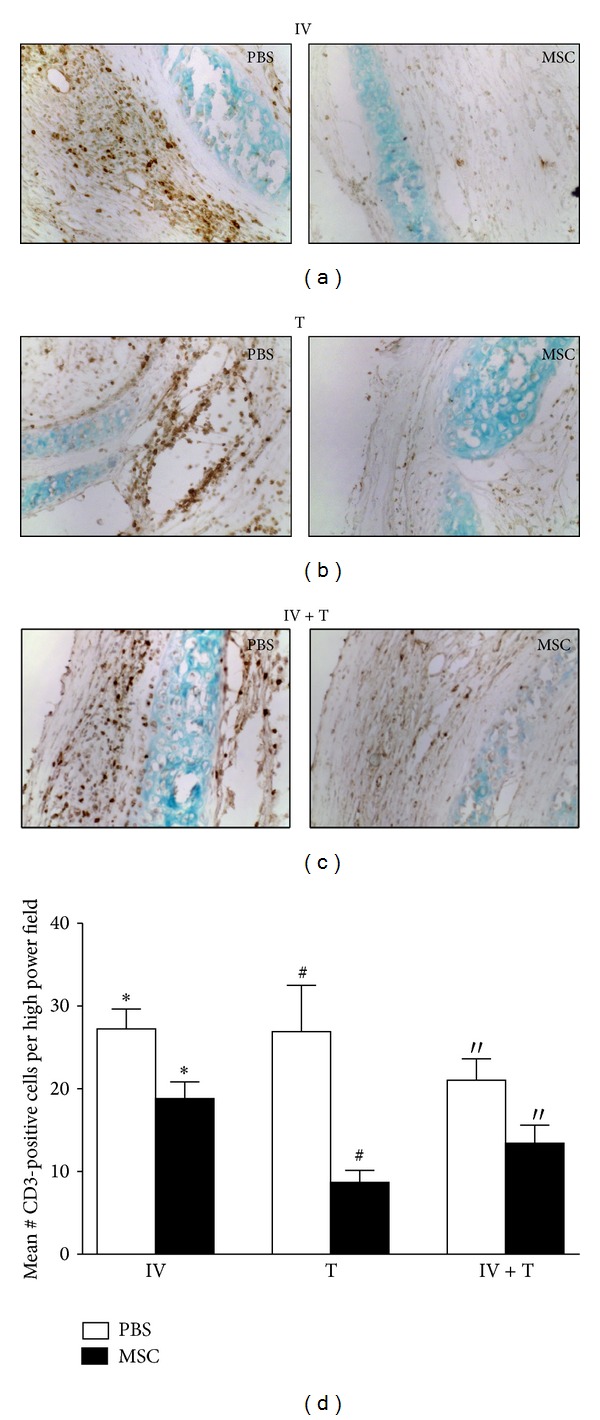
MSCs inhibit T-cell response in the allografts. To assess T-cell response, grafts from each treatment group were explanted 4 weeks after transplantation; IV only (PBS, *n* = 5; MSC, *n* = 6), T (PBS, *n* = 6; MSC, *n* = 6), or IV + T group (PBS, *n* = 4; MSC, *n* = 5). (a)–(c) Tracheal sections stained with anti-CD3 antibodies (dark brown spots) showed significantly less numbers of infiltrated CD3-positive cells in the MSC/IV-treated group (a), MSC/T-treated group (b), and MSC/IV + T-treated group (c) at 4 weeks after transplantation when compared to the PBS-control group (all representative images 200x original magnification). (d) CD3-positive cells were counted in 4 random high power fields per section. Mean counts of cells 4 weeks after transplantation in each MSC treated group (IV only, T only, IV + T) were compared to the respective PBS-control group. Significantly fewer CD3-positive cells were seen in all MSC-treated groups at 4 weeks in IV group (*P < 0.05), T group (^#^ *P* < 0.01), and IV + T group (′′*P* < 0.05). All error bars in (d) indicate SEM.

T- and B-lymphocytes are seen at the site of injury after innate immune cells have encroached into the allograft. Independent from the route of delivery, significantly fewer CD3-positive T-cells were seen in all MSC-treated groups, that is, IV, T, and IV + T at 4 weeks after transplantation (Figure 4). At other time points (2 days, 1 week, and 2 weeks), however, there was no significant difference in the number of CD3 positive cells between MSC-treated and PBS-control groups (data not shown). These data suggest that MSCs could reduce T-cell response in the allografts independent from the route of delivery.

### 3.4. Less Fibrosis in MSC-Treated Recipients

**Figure 5:**
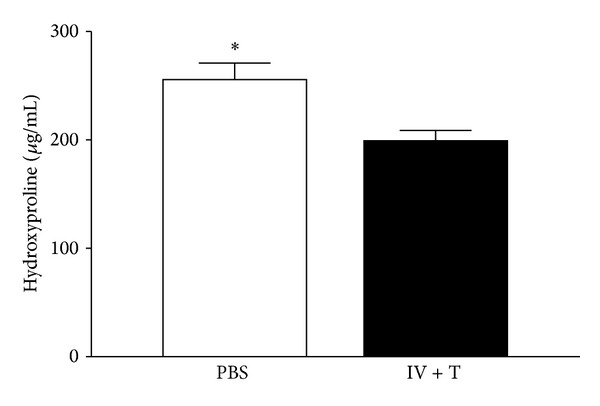
Less fibrosis in MSC-treated recipients. Hydroxyproline-based quantification of collagen after 4 weeks in tracheal transplants from PBS-control (*n* = 6) and MSC/IV + T-treated (*n* = 6) group. MSC/IV + T-treated mice had significantly less collagen deposition 4 weeks after transplantation (*P* = 0.01). All error bars indicate SEM.

During disease course a repair response is initiated involving fibroblast proliferation and extracellular matrix deposition [5]. To investigate whether administration of MSC has beneficial effects on the development of fibrosis, hydroxyproline content in MSC-treated tracheas was analyzed. We found significantly lower hydroxyproline concentrations at 4 weeks after transplantation in the IV + T group (Figure 5). There was no significant difference in the IV + T group at 2 days, 1 week, and 2 weeks after transplantation (data not shown). Once again, similar to what we found with the neutrophils and macrophages, no significant difference in hydroxyproline content was seen in the MSC/IV- or MSC/T-treated groups at any time points (data not shown). These data suggest that MSCs delivered via IV + T could prevent fibrosis in the allografts 4 weeks after trachea transplantation.

### 3.5. MSCs Exert Allograft Protection in a Dose Independent Manner

When MSCs were delivered through combination of intratracheal (1 × 10^5^ cells) and intravenous (1 × 10^5^ cells) routes, two times the amount of MSCs were given to the recipient mice. We designed experiments to rule out the possibility that the greater beneficial effect of the MSC/IV + T-treatment was achieved because of the higher dose of MSCs given. In the subsequent allograft transplantations, 2 × 10^5^ MSCs and 10 × 10^5^ MSCs were injected either intratracheally or intravenously. Comparison of Ly6C/6G-positive cell (neutrophil) numbers showed no difference between 1 × 10^5^ MSC-treated and 2 × 10^5^ cells or 10 × 10^5^ MSC-treated mice (data not shown). These results indicated that MSCs exerted allograft protection in a dose independent manner.

### 3.6. Immunomodulation by MSCs Does Not Require Epithelial Engraftment

**Figure 6:**
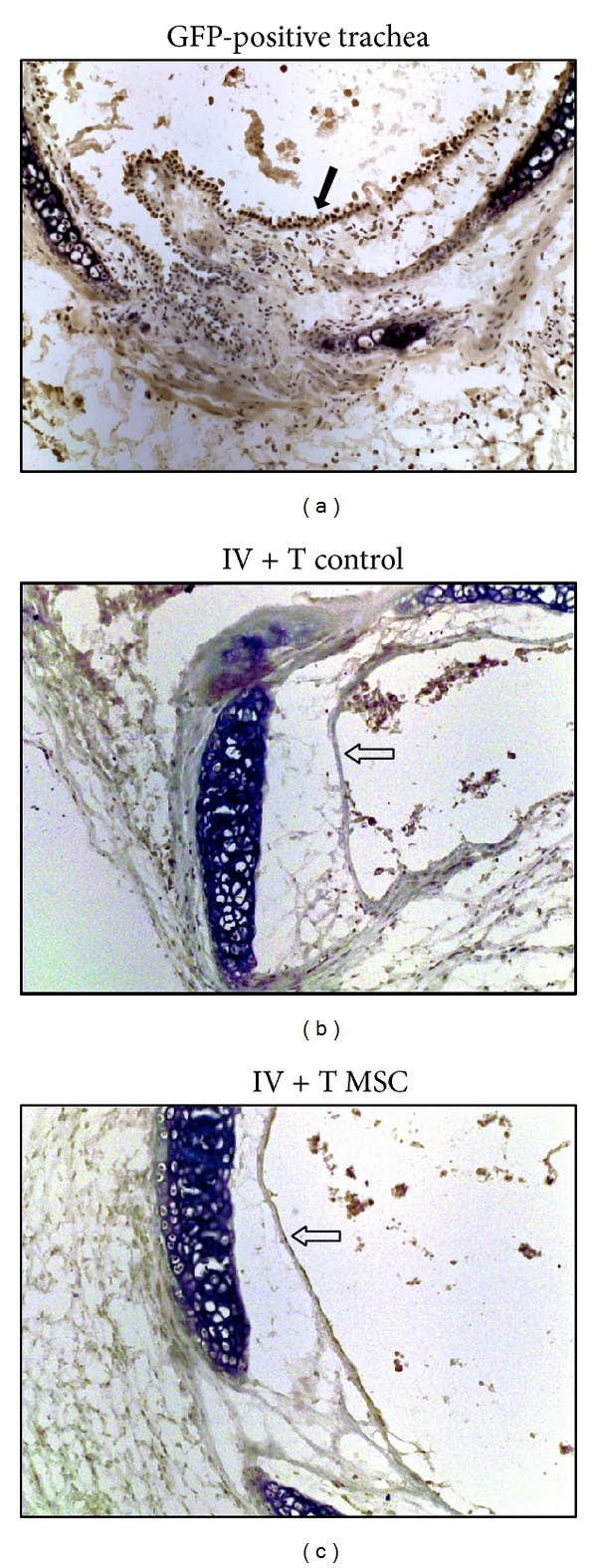
Immunomodulation by MSCs does not require epithelial engraftment. Immunohistochemical staining was performed to determine epithelial engraftment of GFP-MSCs in transplanted trachea. (a) shows positive GFP-staining of tracheal epithelium (brown color indicated by a solid arrow) and parenchymal cells of a trachea section from GFP-mouse. (b) shows negative GFP-staining of tracheal epithelium (indicated by an empty arrow) from IV + T control group. (c) shows absence of GFP-MSCs on tracheal epithelium (indicated by an empty arrow) from IV + T MSC-treated group.

We also performed tracheal allograft transplantation experiments using GFP-labeled MSCs. We found that MSCs given locally or systemically had no significant integration in the tracheal epithelium (Figure 6). This result suggested that epithelial engraftment may not be a prerequisite for the immunomodulation by MSCs observed in this study. Additionally the MSCs do not seem to contribute to new epithelial cells in the tracheal allografts.

### 3.7. Lack of Improvement in Luminal Obliteration or Epithelial Integrity

**Figure 7:**
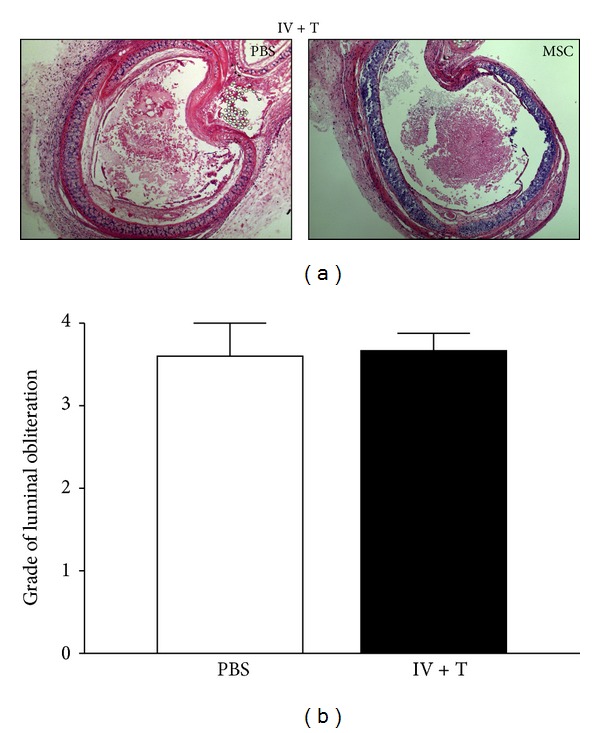
No significant changes in tracheal luminal obliteration. MSC/IV + T-treated group ((a) representative image 40x original magnification) and PBS-control group were explanted 4 weeks after transplantation. Histological grading of H&E stained samples did not show significant differences in median obliteration when compared to PBS-control groups (b). Likewise, no MSC/IV-treated group or MSC/T-treated group showed any difference in luminal obliteration (data not shown).

Development of OB is characterized by loss of epithelium in the initial phase after transplantation and subsequent fibroproliferative processes that lead to luminal obliteration of the airways. We investigated whether MSCs could prevent or slow down these processes. As shown in Figure 7, the degree of luminal obliteration of tracheal allografts was comparable in all cases between MSC-treated and PBS-control groups. Similarly, histopathologic evaluation of luminal integrity of the trachea of MSC-treated recipients did not show significant improvement when compared to the control group (data not shown). The lack of improvement in luminal obliteration or epithelial integrity seems to be consistent with the lack of MSC engraftment of the tracheal allografts described above.

## 4. Discussion

Lung transplantation is the only available treatment option for many end-stage pulmonary diseases. However, long-term allograft survival in lung transplantation is hampered by chronic graft dysfunction. Major improvements in surgical techniques, management with immunosuppressive agents, and control of infections have improved 1-yr survival to 70–80%, but mortality due to OB remains alarmingly high, with only 40–50% survival five years after OB develops [5, 9, 33, 34]. The lung has the highest rate of rejection among all solid organ transplants, probably owing to epithelial immunological vulnerability and injury due to its constant exposure to airborne antigens, pathogens, and pollutants. Although the pathophysiology leading to OB is not fully understood, immune response against donor antigens plays a key role in the disease development. Experiments using a heterotopic tracheal transplantation model demonstrate that alloimmunity is required [7]. Currently available therapeutics have little efficacy. Consequently, OB remains a daunting challenge with limited treatment options and unacceptably high mortality. Thus, continued search for novel strategies to reduce chronic graft dysfunction after lung transplantation is warranted.

Cell-based regenerative therapies for lung diseases offer great promise, including application of the widely studied MSCs [20]. While it was initially thought that transplanted MSCs would engraft and migrate to the sites of injury to replace dysfunctional cells and interact with inflammatory cells, accumulating evidence suggests that the majority of systemically administered MSCs are trapped in capillary networks, for example, the pulmonary first-pass effect, and have a short life span, and distribution to other organs in the body is transient and negligible [35–38]. In the current study, we investigated the effects of MSCs in a heterotopic tracheal transplantation model. Our findings show that treatment of recipient mice with MSCs suppressed neutrophil, macrophage, and T-cell infiltration and reduced fibrosis supporting the critical role of MSCs in limiting tissue injury by modulating the immune responses. The observation that the beneficial immunomodulatory effects of MSCs did not require epithelial engraftment suggests paracrine effects of the cells. When stimulated by injury and inflammation, MSCs likely release a number of soluble factors which may exert a net trophic effect on tissue, stimulate angiogenesis, limit cellular apoptosis and recruit immune cells to the site of injury, and ultimately reduce fibrosis. Each of these effects can occur independently of MSC engraftment and differentiation [39]. Earlier studies investigated MSCs in heterotopic tracheal transplantation model [26, 40], but epithelial integration has not been examined. Furthermore, the most effective method of MSC administration is unknown and particularly important when considering future clinical application. Our study indicates that combination of local and systemic delivery of MSCs more effectively ameliorates the development of inflammation in obliterative airway disease, which suggests that local as well as systemic immunomodulation is important. In addition, our study show that the MSCs did not engraft nor did they seem to contribute to new epithelial cells. Since improved epithelial integrity prevents luminal obliteration after tracheal injection of epithelial/progenitor cells [41], lack of epithelial integration of MSCs might explain the lack of improvement in luminal obliteration in our experiments.

While clinical studies have just begun to examine the effectiveness of MSCs in the prevention of solid organ allograft rejection (ClinicalTrials.gov identifier NCT01668576: Properties of Mesenchymal Stem Cells in Lung Transplant), abundant evidence from animal models suggests that this approach may have merit. In the majority of these investigations, allogeneic MSCs were coinfused at the time of organ transplantation. Improved allograft survival and reduction in the need for concurrent pharmacological immunosuppression have been reported [42–44]. Not surprisingly, the potent immunomodulatory effects of MSCs on T-cell response appear to be of primary importance in their ability to prevent allograft rejection. Interestingly, in the current study MSCs exerted allograft protection in a dose independent manner, the underlying mechanism of which warrants further investigation. Previous studies with genetic lineage tracing and selective ablation of epithelial cells indicate the presence of lung progenitor cells [19, 20]. The difficulties to expand these putative lung progenitors adequately post a major challenge for their therapeutic application. A recent report suggests that injection of recipient epithelial progenitors prevents epithelial loss and decreases OB development [41]. Thus, a combination of MSCs and lung epithelial progenitors may represent a rational proposition for future experimentation, where epithelial progenitors preserve luminal integrity and MSCs attenuate inflammation. A multipronged treatment may be the best way to approach a complex problem of rejection in the immunogenic milieu of the lung that is constantly exposed to triggers of inflammation. A multitude of desirable properties of MSCs may render these cells useful as a therapeutic modality for variety of conditions including lung transplantation. Further efforts on elucidating the molecular mechanisms of MSCs' beneficial effects will help us understand the most effective ways to use these cells clinically.
